# The relationship between sleep disturbance and obsessive– compulsive symptoms: the mediation of repetitive negative thinking and the moderation of experiential avoidance

**DOI:** 10.3389/fpsyg.2023.1151399

**Published:** 2023-07-05

**Authors:** Xudong Zhao, Liao Shen, Yufei Pei, Xiaojun Wu, Ningning Zhou

**Affiliations:** ^1^Department of Psychiatry, Huzhou Third Municipal Hospital, The Affiliated Hospital of Huzhou University, Huzhou, China; ^2^Department of Psychiatry, The First Affiliated Hospital, Zhejiang University School of Medicine, Hangzhou, China; ^3^Shanghai Key Laboratory of Mental Health and Psychological Crisis Intervention, Affiliated Mental Health Center (ECNU), School of Psychology and Cognitive Science, East China Normal University, Shanghai, China; ^4^Department of Clinical Psychology, Huzhou Third Municipal Hospital, The Affiliated Hospital of Huzhou University, Huzhou, China

**Keywords:** sleep disturbance, repetitive negative thinking, obsessive-compulsive symptoms, experiential avoidance, mediation, moderation

## Abstract

**Background:**

Studies have found that sleep disturbance is associated with obsessive–compulsive symptoms. This study aimed to elaborate on the mediating and moderating mechanisms between these two variables. We hypothesized that repetitive negative thinking plays a mediating role in the relationship between sleep disturbance and obsessive–compulsive symptoms, and experiential avoidance plays a moderating role.

**Method:**

This study included 639 Chinese adults. A questionnaire survey was used to assess sleep quality, obsessive–compulsive symptoms, experiential avoidance, repetitive negative thinking, and depression symptoms. A moderated mediation model was established.

**Results:**

After controlling for depressive symptoms, repetitive negative thinking partially mediated the positive correlation between sleep disturbance and obsessive–compulsive symptoms. This indirect relationship was significant in individuals with lower experiential avoidance levels. Particularly, the relationship between sleep disturbance and repetitive negative thinking was significant among individuals with lower experiential avoidance levels, but not among individuals with higher experiential avoidance levels.

**Conclusion:**

This study demonstrated that repetitive negative thinking partially mediated the impact of sleep disturbance on obsessive–compulsive symptoms. The findings suggest that when providing support to individuals with sleep disturbance and obsessive–compulsive symptoms, assessing their level of experiential avoidance is necessary for performing targeted interventions. Individuals with low experiential avoidance may benefit from a clinical intervention targeting repetitive negative thinking to improve sleep quality and obsessive–compulsive symptoms.

## Introduction

Sleep is a vital psychobiological process related to physical and mental health. Sleep disturbances are often accompanied by certain psychiatric disorders. A review of cross-sectional and longitudinal studies found a positive relationship between sleep difficulties and anxiety symptoms among community and clinical participants, including children and adolescents (Willis and Gregory, 2015). Notably, a number of studies have identified a significant positive association between sleep problems and obsessive–compulsive symptoms (Paterson et al., 2013; Nota and Coles, 2015; Cox and Olatunji, 2016). Eveningness gradually influenced the progress of obsessive–compulsive symptoms (OCS) to some degree in an adult clinical sample due to its impact on sleep disturbance (Cox et al., 2018a). Sleep disturbance levels were correlated with OCS, even after controlling for depression among 2071 participants in a national representative survey of English-speaking adults in the United States (Cox and Olatunji, 2016). In addition, a case study indicated that cognitive behavioral therapy with adjunctive chronotherapy was beneficial for treating OCD patients with elevated rates of delayed sleep phases (Coles and Sharkey, 2011). An intervention study with a pediatric sample demonstrated that sleep problems at baseline interfered with the effectiveness of cognitive behavior therapy in reducing OCS symptoms (Ivarsson and Skarphedinsson, 2015). Overall, this indicates that sleep disturbance may aggravate OCS and impede its recovery, where interventions for sleep disturbance may decrease OCS. However, we should notice that higher OCD symptom severity were was significantly associated with more depressive symptoms in a clinical sample, so depression needs to be controlled when examining the relationship between sleep disturbance and OCS (Browning et al., 2021).

Previous research mainly focused on exploring whether sleep disruption is related to OCS. However, the underlying mechanisms remain unclear. As sleep disturbances commonly result in dysfunctional cognitive processes, such as repetitive negative thinking (RNT; Nota and Coles, 2015; Collet et al., 2020) and OCS is associated with these cognitive processes according to a meta-analysis (Norman et al., 2019), these cognitive processes may be crucial for understanding the relationship between sleep disturbance and OCS. Therefore, this study aimed to examine the role of dysfunctional cognitive processes, namely, RNT, in the relationship between sleep disturbances and OCS. Furthermore, the condition under which sleep disturbance is related to OCS was investigated. Experiential avoidance was considered the moderator, based on its moderating effect on some mental health problems (Andrew and Dulin, 2007; Levin et al., 2018).

### Mediating role of RNT

RNT refers to a sustained and abstract focus on the negative individual experiences that are difficult to restrain (Watkins, 2008). It is a cognitive emotion regulation strategy and an established transdiagnostic phenomenon associated with anxiety and mood psychopathology (Ehring and Watkins, 2008; McEvoy et al., 2010). Sleep disturbances may aggravate RNT. A cross-sectional study with 1,021 adolescents from a public school district found that sleep problems were linked to RNT, such as rumination, obsessions, and post-event processing (Stewart et al., 2020). Another cross-sectional study reported that poor sleep quality was positively correlated with worry and rumination among adolescents (Lin et al., 2019). A longitudinal study indicated that chronic sleep problems had a causal effect on RNT presented as obsessions and rumination through inhibitory control (Cox et al., 2018b). Another longitudinal study found that insomnia symptoms increased RNT through executive and emotional regulatory functions (Cox et al., 2019).

RNT was found to be associated with emotional disorders, including OCD (Ehring and Watkins, 2008), and there was a positive correlation between RNT and OCS (Arditte et al., 2016). The relationship between RNT and OCS is associated with a lack of inhibitory control (Nota et al., 2016). Impairments in inhibitory control can lead to complex cognitions and behaviors, including repetitive intrusive thoughts, repetitive negative thinking, and excessive perseverative behaviors that define OCS (Nota et al., 2016). RNT may refer to the metacognitive, and metacognitive beliefs associated with obsessive–compulsive symptoms (Wells and Papageorgiou, 1998), further suggesting the relationship between RNT and OCD. In a study with 95 OCD patients, participants who received group metacognitive therapy (MCT) improved significantly more than those who received CBT (Papageorgiou et al., 2018). In addition, deterioration rate was lower with MCT than behavioral activation (BA) for other outpatients (Schaich et al., 2023).

Therefore, sleep disturbance may impact OCS through RNT. That is, sleep disturbance may perturb the capacity to inhibit distractors for the sake of focusing on the desired stimulus, which may facilitate RNT and further confer vulnerability to OCS.

### Moderating role of experiential avoidance

Although researchers have empirically established that sleep disturbance is related to various psychological symptoms, little attention has been paid to the moderators of these effects. Therefore, examining variables that may regulate the link between sleep disturbance and adverse psychological outcomes is crucial. This study, proposed that experiential avoidance is a critical moderator and investigated whether the extent of individuals’ experiential avoidance caused variations in direct and indirect pathways. Experiential avoidance is characterized by an inclination to avert or inhibit personal thoughts and feelings. Experiential avoidance is a manifestation of an individual’s unwillingness to accept their feelings and thoughts, causing them to attempt to avoid disliked events and thoughts.

An empirical study (Spinhoven et al., 2017) and review (Brereton and McGlinchey, 2020) demonstrated the risky role of experiential avoidance in the etiology, development, and alteration of a variety of psychopathology, including depression, worry, OCS, posttraumatic stress disorders, and self-harm. Researchers found that experiential avoidance moderated psychological distress (Pickett et al., 2011; Bardeen et al., 2013; Bardeen, 2015; Levin et al., 2018; Williams et al., 2019). Specifically, Bjornsson et al. (2010) reported that experiential avoidance moderated the relationship between rumination and depression by demonstrating that rumination was only related to depressive symptoms with high experiential avoidance. Williams et al. (2019) found that experiential avoidance regulated the relationship between behavioral inhibition system susceptibility and prolonged grief disorder. In particular, the correlation between the above two was significant with high experiential avoidance. Additional cross-sectional and longitudinal studies reported an interaction between anxiety sensitivity and experiential avoidance by demonstrating that the correlation of anxiety sensitivity with perceived stress was significant among individuals with high experiential avoidance (Bardeen et al., 2014). As high experiential avoidance may increase the negative effect of some psychological variables on psychopathology, it may serve as an added element to the potential hazards of sleep disturbance. However, some studies reported a differential pattern of experiential avoidance’s moderation effect by demonstrating a significant positive association between anxiety sensitivity and perceived stress at low, but not high, levels of experiential avoidance (Bardeen et al., 2013). Based on the above research, we speculated that the relationship between sleep disturbances and OCS via RNT would be significant among individuals with high experiential avoidance.

In summary, this study examined the mechanism underlying the relationship between sleep disturbances and OCS. Sleep disturbance was anticipated to be linked to higher levels of OCS through RNT (Hypothesis 1). We further explored whether the indirect relationship is regulated by experiential avoidance (Hypothesis 2). Specifically, mediation was expected in individuals with high experiential avoidance.

## Materials and methods

### Participants

Participants were recruited through social media, such as WeChat, advertisements, and online forums. The inclusion criteria were: being aged 18–75 years and having no severe mental disorders such as suicidal tendencies, and schizophrenia, or severe physical illnesses. The participants received 10 RMB as compensation. This study recruited 721 adults, of which 82 were excluded due to completing less than half of the survey. The final sample consisted of 639 participants. Their mean age was 24.88 years, ranging from 18 to 64 years. Most participants were female (67.8%). Demographic information is shown in Table 1.

**Table 1:** Sociodemographic information of the participants (N = 639).

Demographic variables	
Age	24.88 (6.67)
Range	18–64
Gender	
Male	206 (32.2%)
Female	433 (67.8%)
Education level	
Below middle school	47 (7.4%)
Above college	527 (82.4%)
Above master	65 (10.2%)
Occupation	
University students	318 (49.8)
Full time job	283 (44.3%)
Other	38 (6%)
Marital status	
Single	327 (51.2%)
In relationship	312 (48.8%)

### Measurement

#### Obsessive compulsive inventory-revised

Distress related to the multiple dimensions of OCD was assessed using the Obsessive Compulsive Inventory-Revised (OCI-R; Foa et al., 2002). This measure consists of six subscales: ordering, washing, hoarding, checking, obsession, and neutralizing. The validity and reliability of the scale were confirmed (Peng et al., 2011; Chasson et al., 2013; Tang et al., 2015). The Cronbach’s α coefficient of the OCI-R in the current sample was 0.955, demonstrating satisfactory internal consistency reliability. A cut-off score of >20 was considered appropriate to determine OCD (Zhang et al., 2020).

#### Pittsburgh sleep quality index

Subjective sleep quality was evaluated using the Pittsburgh Sleep Quality Index (PSQI), which includes nine self-rated questions with 18 items (Buysse et al., 1989). Several dimensions are used to assess sleep quality, such as onset latency, duration, and efficiency. Each dimension adopts a 0–3 scoring system. Scores range from 0 to 21, with lower scores indicating better sleep quality. The reliability and validity of the Chinese version of the PSQI is good (Liu et al., 1996), and the internal consistency reliability was adequate in the current sample (Cronbach’s α = 0.739). A cut-off score of >7 was confirmed appropriate to determine poor sleep (Zhang et al., 2020).

#### Acceptance and action questionnaire – II

The Acceptance and Action Questionnaire (AAQ) was developed by Bond et al. (2011). This study utilized the second edition of the AAQ (AAQ-II). The scale contains seven items rated on a seven-point Likert scale (1 = never; 7 = always). Scores range from 7 to 49, with higher scores indicating higher experiential avoidance tendency. Cao et al. (2013) translated this scale into Chinese version and tested its validity and reliability, and the result is good. The internal consistency was good in the present sample (Cronbach’s α = 0.901).

#### Repetitive thinking questionnaire

The Repetitive Thinking Questionnaire comprises 10 items (McEvoy et al., 2010) modified from the Rumination Response Scale (Items 1, 3, 10), Penn State Worry Questionnaire (Items 5, 6, 8, 9), and the Post-event Processing Questionnaire-Revised (Items 2, 4, 7). Scores range from 10 to 50 (or 9–49 for the nine items). The RTQ-10 has demonstrated good reliability and validity, and the Chinese version has been validated in a sample of Taiwanese university students (Hu, 2015). The internal consistency of the present sample was good (Cronbach’s α = 0.897).

#### Depression subscale of the depression anxiety stress scale

The Repetitive Thinking Questionnaire comprises (DASS) is used to describe negative emotional experiences or physical reactions in the past week (Akin and Çetin, 2007), with responses rated on a four-point Likert scale (0 = Did not apply to me at all; 3 = Applied to me very much or most of the time). This measure consists of three subscales targeting depression, anxiety, and stress. Each subscale consists of seven items. The present study used the seven items of the Depression subscale. The total score ranges from 0 to 21. The revised Chinese version has good reliability and validity (Gong et al., 2010). The internal consistency reliability was good in the current sample (Cronbach’s α = 0.878).

#### Ethics

The ethics committee of East China Normal University approved all procedures (No. HR1-1047-2020).

### Statistical analysis

SPSS was used to analyze the data, PROCESS, a freely available SPSS computational tool (Hayes, 2013), was utilized to test the potential mediation effect of RNT and moderating role of experiential avoidance in the relationship between sleep disturbance and OCS.

The total missing values were less than 0.01%, and the information loss was less than 0.1% for each participant. Data were imputed using the average value of each scale.

The normal distribution of the data was examined using the Kolmogorov–Smirnov test, and all variables exhibited a normal distribution. Descriptive statistics for each variable were expressed as mean scores, and standard deviations. Pearson correlation coefficients were used to explore the associations among sleep disturbance, experiential avoidance, RNT, and OCS when controlling for depression.

The mediating role of RNT between sleep disturbances and OCS was examined using Model 4 of PROCESS. Bias-corrected bootstrap tests were conducted to measure the significance of the indirect effects with 95% CI. Depression was regarded as a covariate.

The moderating effect of experiential avoidance was tested using Model 59 of PROCESS. The model examined whether direct and indirect effects varied at different levels of experiential avoidance. Depression was a covariate.

## Results

### Descriptive statistics of the main variables

Among the participants, 35.8% of those with sleep disturbances exceeded cut-off scores. 42.1% of those with obsessive–compulsive symptoms exceeded cutoff scores.

The means and standard deviations for the study variables of interest and partial correlations are presented in Table 2. Sleep disturbance was positively correlated with RNT, experiential avoidance, and OCS. RNT was positively correlated with experiential avoidance and OCS. Experiential avoidance was positively associated with OCS.

**Table 2:** Descriptive statistics and partial correlations between study variables after controlling for depression symptoms.

Variables	Means (SD)	Sleep disturbance	Experiential avoidance	Repetitive negative thinking
Sleep disturbance	6.86 (3.29)	1		
Experiential avoidance	26.62 (8.28)	0.22***	1	
Repetitive negative thinking	27.99 (8.52)	0.16***	0.52***	1
OC symptoms	19.60 (14.16)	0.12***	0.22***	0.21***

OC symptoms = obsessive compulsive symptoms.

*** *p* < 0.001.

### Mediation analysis

**Figure 1:**
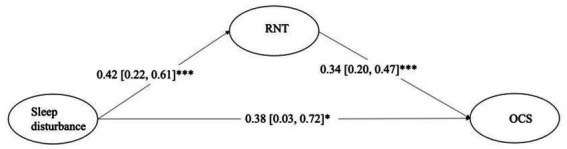
Mediation model indicating the effects of sleep disturbance on obsessive–compulsive symptoms through repetitive negative thinking. ^***^p < 0.001, ^**^p < 0.01, ^*^p < 0.05. RNT = repetitive negative thinking. OCS = obsessive–compulsive symptoms. Depression symptoms were controlled in the analysis.

The results indicated that sleep disturbance had a direct relationship with OCS (β = 0.38, 95% CI [0.03, 0.72]) and an indirect relationship with OCS through RNT (β = 0.14, 95% CI [0.06, 0.24]). Figure 1 presents the detailed information.

As this cross-sectional study could not reveal causal relationships, we did an explorative analysis of the relationship between RNT and OC symptoms via sleep disturbance as a reference. The results demonstrated that the mediation effect was significant. The direct association coefficient between RNT and OC symptoms was 0.34 (0.20, 0.47). The correlations between RNT and sleep disturbance was 0.06 (0.03, 0.09), and that between sleep disturbance and OC symptoms was 0.38 (0.03, 0.72). The indirect effect was 0.02 (0.0005, 0.05).

### Moderate mediation analysis

**Figure 2:**
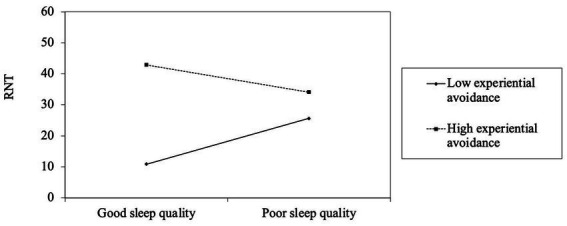
The moderating role of experiential avoidance in the relationship between sleep disturbance and repetitive negative thinking. RNT = repetitive negative thinking. Depression symptoms were controlled in the analysis.

The results indicated that sleep quality was positively and insignificantly correlated with OCS (β = 1.03, 95% CI [−0.13, 2.19]). Sleep quality was significantly positively correlated with RNT (β = 0.93, 95% CI [0.39, 1.47]), and the interaction term of sleep quality and experiential avoidance significantly impacted RNT (β = −0.03, 95% CI [−0.05, −0.01]). The association between sleep quality and RNT was significant with low levels of experiential avoidance (-1SD; β = 0.39, 95% CI [0.15, 0.64]) but not high (+1SD: β = −0.03, 95% CI [−0.24, 0.17]; see Figure 2).

**Figure 3:**
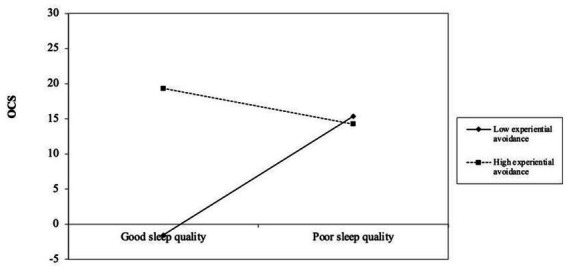
The moderating role of experiential avoidance in the relationship between sleep disturbance and obsessive–compulsive symptoms. OCS = obsessive–compulsive symptoms. Depression symptoms were controlled in the analysis.

The interaction term of sleep quality and experiential avoidance did not significantly influenced OCS (β = −0.03, 95% CI [−0.06, 0.01]). However, the association between sleep disturbance and OCS was significant with low experiential avoidance (-1SD; β = 0.53, 95% CI [0.02, 1.04]) but not high (+1SD; β = 0.14, 95% CI [−0.26, 0.54]; see Figure 3).

The product terms of RNT and experiential avoidance had no significant influence on OCS (β = −0.002, 95% CI [−0.02, 0.01]).

The conditional indirect effect was significant with low experiential avoidance (-1SD; β = 0.09, 95% CI [0.01, 0.19]) but not high (+1SD; β = −0.01, 95% CI [−0.06, 0.04]).

## Discussion

This study shed light on the pathogenesis through which sleep disturbance is associated with OCS by showing that RNT significantly mediated the correlation between sleep disturbance and OCS. Moreover, the indirect relationship was stronger among participants with lower experiential avoidance.

The prevalence of people who met the cutoff criteria of insomnia was comparable to that of previous studies, such as a systematic review that found that the proportion of insomnia during the COVID-19 pandemic was as high as 39.1%, with 37.0% in the early stage and 41.8% in the late stage (Li et al., 2022). The prevalence of people who met the cutoff criteria of OCD was higher than that reported by previous studies (Foa et al., 2002; Tang et al., 2015). The average score of OCI-R was higher than that of college students in China (M = 9.90, SD = 9.31; Tang et al., 2015), but was comparable to those in other studies: M = 18.82, SD = 11.10 (Foa et al., 2002) and M = 28.0, SD = 13.53 (AlHusseini et al., 2021). There are three reasons for this. First, the subjects were recruited at the end of 2020 when the COVID-19 pandemic may have an impact on the OCS in the general population. As is shown in AlHusseini et al.’s (2021) survey, 62.4% of 2,186 people 21 cutoff score in OCI-R. Second, different from Tang et al.’s (2015) study which employed college students as the subjects, most participants in our study were working people. Thirdly, our recruitment advertisement is about sleep. People with sleep disturbances may have been more willing to participate in our study, and these people may have had higher obsessive–compulsive symptoms.

Consistent with previous studies (Nota and Coles, 2015; Cox and Olatunji, 2016), sleep disturbance was directly related to OCS in this sample, even after controlling for depression. RNT mediated this relationship, supporting Hypothesis 1. A possible explanation is that poor sleep leads to a decline in the ability to restrain RNT, and excessive invasion of RNT causes vulnerabilities for the development of psychopathology, such as OCD, as demonstrated in a previous review (Leyro et al., 2010). Although researchers have suggested that sleep disruption aggravates RNT, which leads to negative psychological consequences (Nota et al., 2016), no studies have tested this hypothesis. The present findings demonstrated that sleep disturbance contributed to OCS via the transdiagnostic process of RNT. However, further prospective studies are needed to verify this. These results have considerable implications for the precautions against and therapy for OCD. Sleep could be a crucial target for treating OCD. Regaining good sleep could enhance cognitive control and consequently strengthen the ability to regulate RNT and decrease OCS. Furthermore, as the relevance of RNT for OCD, interventions, such as metacognitive therapy, targeting RNT might be helpful.

The study showed that experiential avoidance played a moderate role in the above relationship. The impact of sleep disturbance on the psychological consequences of RNT and OCS disappeared with high experiential avoidance. These results were consistent with one study (Bardeen et al., 2013) but contradicted other studies (Bjornsson et al., 2010; Pickett et al., 2011). A possible reason is that individuals who choose to avoid their inner experience appear more likely to perceive their RNT and OCS as stressful and unbearable; thus, they may be overwhelmed by RNT and OC symptoms independent of their sleep quality. However, for those lower in experiential avoidance, the negative impacts of sleep disturbance on repetitive negative thinking and obsessive–compulsive symptoms may be evident. The results indicated a diverse pattern of the moderate effect of experiential avoidance between psychopathological variables. Although a number of studies found significant relationships among psychopathological variables under high levels of experiential avoidance (Bjornsson et al., 2010; Pickett et al., 2011), some studies indicated the opposite results. For example, Bardeen et al. (2013) found that experiential avoidance moderated the relationship between anxiety sensitivity and perceived stress, and anxiety sensitivity shared a significant positive association with perceived stress at low, but not high, levels of experiential avoidance. Pickett and Kirby (2010) demonstrated individuals with lower experiential avoidance scores exhibited a bias towards activating positive emotion inferences, which indicated the moderated role of EA in the emotional processing. As this may be the first study to examine the interactive relationship between experiential avoidance and sleep disturbances, the conclusion requires further examination, and a follow-up study is needed.

The present findings could assist in future intervention development. Sleep-related interventions may provide better responses by tailoring elements for those with low experiential avoidance among OCD patients. Among individuals with high experiential avoidance, sleep disturbance did not show a statistically significant or clinical relationship with RNT or OCS, suggesting that sleep intervention among this subpopulation may be ineffective or should be performed after their levels of experiential avoidance decrease. In contrast, significant direct and indirect effects among individuals with low experiential avoidance indicate a further need for intervention to remove the effects of sleep disturbance on RNT and OCS. Therefore, for OCD patients with varying levels of experiential avoidance, the influence of sleep disturbance on OCS differs, and intervention elements should be modified.

Furthermore, RNT mediates the sleep disturbance and OCS in people’s daily life. For instance, when people have difficulty falling asleep, they often engage in repetitive negative thinking, such as, “If I cannot fall asleep right now, I will have insomnia tonight and will not have energy tomorrow.” This type of thinking may result in a vicious circle and generate compulsive thinking and obsessive–compulsive behavior, which may lead to OCS and even OCD.

This study had some limitations. First, cross-sectional data were used to conduct this research; therefore, causal inferences could not be made. It remains unclear whether unhealthy sleep results in more severe OCS via RNT, as the opposite direction is possible. We did an explorative analysis of a mediation model with sleep disturbance (M) mediating the OC symptoms (Y) in relation to RNT (X), and the results showed that the mediation model is valid. Prospective studies should be longitudinal or interventional to examine causal relationships. Second, this study relied exclusively on self-reported information, which may be a limitation for evaluating symptoms through self-assessment. Further studies may benefit from observation, physiological scores aimed at anxiety, and emotion recognition scenarios based on the situation. Moreover, an objective assessment of sleep, such as polysomnography, may be valuable considering the subjective characteristics of the PSQI. Third, the present study omitted biological factors that mediate the relationship between sleep quality and OCS. Previous research has demonstrated a connection between OCS and the cortico-striato-thalamo-cortical circuit (Tang et al., 2016). The thalamus and cortex are involved in conscious awareness and closely linked to human sleep. Therefore, activity in some brain regions may regulate the connection between sleep disturbances and OCS. In addition, environmental factors, such as the COVID-19 pandemic, may be a confounding factor, which increases the risk of OCD (Wheaton et al., 2021; Kroon et al., 2022). Furthermore, the almost half of the participants were college students, and 92% had college education or higher. Therefore, these findings may not generalizable to the general population. The sample should be expanded to other educational level groups in future studies. Moreover, a non-clinical community sample was investigated in this study. Adding clinical samples in future studies would expand practical significance and enrich the clinical value of the findings. Finally, the subtypes of sleep disturbance and OCD should be further examined in future studies.

## Conclusion

This study examined the mediation effect of RNT in the relationship between sleep disturbance and OCS and the moderating role of experiential avoidance in this relationship. When treating patients diagnosed with OCD who have comorbid sleep disturbance, clinical workers should evaluate and focus on experiential avoidance levels. Individuals with low experiential avoidance may benefit from clinical interventions targeting repetitive negative thinking to improve sleep quality and obsessive–compulsive symptoms.

## Data availability statement

The raw data supporting the conclusions of this article will be made available by the authors, without undue reservation.

## Ethics statement

The studies involving human participants were reviewed and approved by Ethics Committee of East China Normal University. The patients/participants provided their written informed consent to participate in this study.

## Author contributions

NZ: conceptualization, methodology, formal analysis, writing –original draft, writing – review and editing, supervision, and project administration. XZ: investigation and writing –original draft. LS: conceptualization, writing – review and editing, and supervision. YP: methodology, formal analysis, writing –review and editing, and supervision. XW: investigation, writing – review and editing, and supervision. All authors contributed to the article and approved the submitted version.

## Funding

This work was supported by the National Social Science Foundation of China (21CSH091), the Fundamental Research Funds for the Central Universities (43800-20101-222104), the Basic Public Welfare Research Program of Zhejiang Province (LTGY23H090013), and Huzhou Municipal Science and Tech Commission (2020GYB52) for the investigation of the study and open access publication fees.

## Conflict of interest

The authors declare that the research was conducted in the absence of any commercial or financial relationships that could be construed as a potential conflict of interest.

## Publisher’s note

All claims expressed in this article are solely those of the authors and do not necessarily represent those of their affiliated organizations, or those of the publisher, the editors and the reviewers. Any product that may be evaluated in this article, or claim that may be made by its manufacturer, is not guaranteed or endorsed by the publisher.
